# Piperine as a Multifunctional Epigenetic Modulator: Integrative Molecular Insights into Cancer and Chronic Disease Therapy

**DOI:** 10.3390/ijms27115149

**Published:** 2026-06-05

**Authors:** Andrés Alarcón, Catherine Meza, Carolina Añazco, Marcela Sepúlveda, Sebastián Alarcón, Sharin Valdivia

**Affiliations:** 1 Cancer Biology Laboratory, Facultad de Medicina, Universidad San Sebastián, Sede Concepción, Campus Tres Pascualas, Concepción 4080871, Chile; alarcon.andres1707@gmail.com; 2 Departamento de Ciencias Biológicas y Químicas, Facultad de Ciencias, Universidad San Sebastián, Sede Concepción, Campus Tres Pascualas, Concepción 4080871, Chile; catherine.meza@uss.cl (C.M.); marcela.sepulveda@uss.cl (M.S.); 3 Nutritional Biochemistry Laboratory, School of Nutrition and Dietetics, Faculty of Rehabilitation and Quality of Life Sciences, San Sebastian University, Valdivia 5091000, Chile; carolina.anazco@uss.cl; 4 Escuela de Medicina, Facultad de Medicina, Universidad San Sebastián, Sede Concepción, Campus Tres Pascualas, Concepción 4080871, Chile

**Keywords:** piperine, epigenetic regulation, DNA methylation, histone deacetylases, microRNAs, redox signaling, cancer stem cells

## Abstract

Piperine, the principal alkaloid of Piper nigrum, has gained attention as a multifunctional dietary compound with broad effects on epigenetic and transcriptional regulation in cancer and chronic diseases. Evidence shows that piperine modulates DNA methylation (↓ DNMT3B), histone acetylation (↓ HDAC activity), and microRNA networks (↑ miR-29c, ↓ miR-383), thereby reshaping key oncogenic and tumor-suppressive pathways. Beyond canonical epigenetic control, it can also stabilize G-quadruplex structures in promoters such as c-MYC, adding an architecture-based mechanism of transcriptional repression. Its dual redox behavior—antioxidant at low doses and pro-oxidant at higher doses—confers context-dependent selectivity, enabling oxidative stress–mediated apoptosis in tumor cells. Compared with other nutriepigenetic agents (curcumin, resveratrol, EGCG), piperine stands out for its multi-target profile and preliminary evidence of activity against cancer stem cell–like phenotypes. Nonetheless, limited solubility, rapid metabolism, and scarce in vivo validation constrain clinical translation. Future efforts should focus on advanced formulations, multi-omics approaches, and cancer stem cell models to better define its therapeutic potential and safety.

## 1. Introduction

Currently, epigenetic regulation is recognized as a central mechanism in the initiation and progression of cancer and other chronic diseases, as it integrates reversible modifications capable of altering gene expression without changing the underlying DNA sequence [1,2,3]. These regulatory layers include DNA methylation, histone modifications, chromatin remodeling, and non-coding RNA networks, all of which dynamically interact with environmental, metabolic, inflammatory, and dietary stimuli. In cancer, epigenetic dysregulation contributes to aberrant transcriptional programs associated with proliferation, therapeutic resistance, cellular plasticity, and tumor progression, while its reversible nature has positioned the epigenome as an attractive therapeutic target.

An additional layer of complexity arises from the close interplay between oxidative stress and epigenetic regulation. Reactive oxygen species (ROS) can influence the activity of DNA methyltransferases (DNMTs), histone deacetylases (HDACs), and TET enzymes, alter the availability of methyl donors and metabolic cofactors, and induce oxidative DNA damage, thereby affecting transcriptional fidelity and chromatin organization [4,5,6]. Conversely, epigenetic programs regulate antioxidant pathways, including NRF2-dependent responses, establishing a bidirectional relationship between redox signaling and gene regulation. This functional crosstalk has gained increasing relevance in cancer biology, where oxidative stress, epigenetic plasticity, and oncogenic signaling frequently converge. Within this integrative framework, dietary bioactive compounds have emerged as potential modulators of epigenetic and transcriptional regulation, giving rise to the field of nutriepigenetics [7,8]. Among these compounds, piperine—the principal pungent alkaloid of *Piper nigrum* and *Piper longum*—has attracted growing attention due to its multitarget biological activity. Structurally, piperine (C_17_H_19_NO_3_) is a hydrophobic alkaloid composed of a piperidine ring linked to a methylenedioxyphenyl group through a conjugated chain, a configuration that may contribute to membrane permeability and interaction with intracellular targets [9,10]. Experimental studies have demonstrated that piperine can influence diverse biological processes associated with cancer progression, including apoptosis, inflammatory signaling, oxidative stress, epithelial–mesenchymal transition, and stemness-related pathways. Despite these promising effects, piperine also presents important pharmacokinetic limitations, including low aqueous solubility, extensive metabolism, and variable bioavailability. Nevertheless, experimental studies have demonstrated systemic absorption, tissue distribution, and interactions with drug-metabolizing enzymes and transporters, suggesting that biologically relevant intracellular concentrations can be achieved under certain conditions [11,12]. However, whether piperine or its metabolites directly access nuclear compartments and epigenetic machinery at sufficient concentrations remains incompletely understood and should be interpreted cautiously. Mechanistically, emerging evidence suggests that piperine may influence multiple epigenetic and transcription-associated pathways. In cancer models, piperine has been reported to modulate microRNA networks, including upregulation of the tumor suppressor miR-29c together with reduction in DNMT3B expression, suggesting a coordinated mechanism of epigenetic reprogramming [13]. Additional studies indicate that piperine may interact with non-canonical DNA structures such as G-quadruplexes within oncogene promoters, including *c-MYC*, although this mechanism remains preliminary and requires broader validation [14]. Beyond epigenetic regulation, piperine also influences key oncogenic signaling pathways, including Wnt/β-catenin, PI3K/AKT/mTOR, NF-κB, and STAT3/Snail-mediated epithelial–mesenchymal transition, which collectively regulate proliferation, invasion, cellular plasticity, and apoptosis [15,16,17]. Given the growing but still fragmented evidence in this field, this review aims to critically examine the role of piperine as a multitarget modulator of epigenetic, transcriptional, and redox-sensitive pathways in cancer and chronic diseases. Particular emphasis is placed on the interaction between DNA methylation, microRNA regulation, oxidative stress, and transcriptional signaling networks, while also discussing current limitations regarding mechanistic specificity, pharmacokinetics, and translational applicability.

## 2. Relevance of Epigenetic Modulation in Cancer and Chronic Diseases

Epigenetic alterations are not merely secondary events in disease progression but are increasingly recognized as early and potentially reversible drivers of pathological phenotypes. In cancer, aberrant DNA methylation, histone modifications, and non-coding RNA dysregulation contribute to the silencing of tumor suppressor genes, activation of oncogenic pathways, and disruption of cellular differentiation programs. Importantly, these epigenetic changes are dynamically influenced by metabolic state, inflammatory signaling, oxidative stress, and environmental exposures, reinforcing the concept of epigenetic plasticity. This reversibility has positioned epigenetic regulation as a high-value therapeutic target, particularly in contexts where genetic mutations alone do not fully explain disease heterogeneity or treatment resistance [1,2,18,19].

In this regard, pharmacological epigenetic therapies such as DNA methyltransferase (DNMT) inhibitors and histone deacetylase (HDAC) inhibitors have demonstrated clinical utility, particularly in hematological malignancies. Agents including azacitidine, decitabine, vorinostat, and romidepsin have shown that epigenetic alterations can be therapeutically targeted and partially reversed [20,21]. However, their limited specificity, dose-dependent toxicities, and variable long-term responses highlight the need for alternative strategies capable of modulating epigenetic pathways in a more controlled and context-dependent manner. Consequently, naturally derived bioactive compounds have gained increasing attention due to their ability to influence multiple regulatory networks associated with apoptosis, oxidative stress, inflammation, and transcriptional regulation. Indeed, several phytochemicals have been reported to modulate microRNAs, histone-modifying enzymes, DNA methylation-associated pathways, and oncogenic signaling cascades involved in tumor progression and therapeutic response [7,8,22]. Within this framework, nutriepigenetics has emerged as a promising but still developing field aimed at understanding how dietary compounds influence gene expression through epigenetic mechanisms. Rather than acting as highly selective enzyme inhibitors, many natural compounds exert pleiotropic and context-dependent effects influenced by concentration, cellular environment, metabolic state, and bioavailability. For example, the miR-29 family is known to regulate DNA methyltransferases including DNMT3A and DNMT3B, thereby establishing functional links between post-transcriptional regulation and epigenetic control of gene expression [22,23,24]. Similarly, several phytochemicals have demonstrated the capacity to influence apoptosis, proliferation, stemness, and differentiation through coordinated modulation of epigenetic and transcription-associated pathways [25,26].

Taken together, these observations highlight the need for a critical and mechanistically grounded evaluation of dietary bioactive compounds, distinguishing well-supported epigenetic effects from preliminary or context-dependent findings. This distinction is particularly relevant for compounds such as piperine, whose biological activity spans multiple regulatory layers—including oxidative stress, apoptosis, microRNA modulation, and transcriptional signaling—yet whose mechanistic integration and direct epigenetic specificity remain incompletely defined.

## 3. Relevance of Epigenetic Modulation in Cancer

Epigenetic dysregulation is now recognized as a central driver of cancer initiation and progression rather than a secondary consequence of genetic alterations. Aberrant DNA methylation patterns, including promoter hypermethylation of tumor suppressor genes and global hypomethylation, contribute to genomic instability and oncogene activation across multiple cancer types [1,2]. In parallel, alterations in histone modification landscapes further reinforce transcriptional reprogramming, enabling the stabilization of malignant phenotypes. Importantly, these epigenetic alterations are functionally linked to the acquisition of stem-like properties in tumor cells. The deregulation of pluripotency-associated factors such as OCT4, SOX2, and NANOG has been associated with the emergence of cancer stem cell (CSC) populations, which play a critical role in tumor recurrence, metastasis, and therapeutic resistance [27,28]. A key feature distinguishing epigenetic alterations from genetic mutations is their reversibility. This has positioned epigenetic regulation as a promising therapeutic target, particularly in contexts where tumor heterogeneity and adaptive resistance limit the efficacy of conventional treatments. However, current pharmacological epigenetic therapies, such as DNMT and HDAC inhibitors, often lack specificity and may induce significant off-target effects. In this context, increasing attention has been directed toward naturally derived compounds capable of modulating epigenetic pathways in a multi-target and context-dependent manner. Nevertheless, the extent to which these compounds exert direct epigenetic effects versus indirect modulation through redox signaling or transcriptional networks remains incompletely understood, highlighting the need for critical evaluation of their mechanisms of action.

## 4. Epigenetic Regulatory Networks in Cancer and Chronic Disease

Epigenetic regulation in cancer and chronic diseases operates through highly interconnected mechanisms involving DNA methylation, histone modifications, non-coding RNAs, and chromatin remodeling. These processes are dynamic and responsive to environmental, dietary, inflammatory, and metabolic stimuli, collectively shaping gene expression programs without altering the underlying DNA sequence [1,2,3,25,29].

Among these regulatory layers, microRNAs (miRNAs) play a central role in post-transcriptional gene regulation. These small non-coding RNAs (20–24 nucleotides) can inhibit mRNA translation or promote degradation, thereby modulating key cellular processes such as apoptosis, proliferation, differentiation, and immune response [30,31,32].

Dysregulation of miRNAs has been widely implicated in disease pathogenesis, with oncomiRs such as miR-21 and miR-155 frequently overexpressed in cancer, while tumor suppressor miRNAs such as miR-34a are often silenced through epigenetic mechanisms, including promoter methylation [33,34,35,36].

In metabolic diseases, altered miRNA expression contributes to dysregulation of key signaling pathways such as PI3K/AKT and AMPK, promoting insulin resistance, lipid imbalance, and progression of cardiovascular complications [37,38].

DNA methylation remains one of the most extensively studied and clinically relevant epigenetic modifications. Aberrant methylation patterns, including promoter hypermethylation of tumor suppressor genes and global hypomethylation, contribute to genomic instability and oncogenic activation. Clinically, DNA methylation has enabled the development of diagnostic tools such as the SEPT9 methylation test for colorectal cancer and the VIM methylation assay in bladder cancer [39,40,41].

Furthermore, pharmacological inhibition of DNA methyltransferases (DNMTs), using agents such as 5-azacytidine and decitabine, has demonstrated therapeutic efficacy in hematological malignancies, although these approaches are often limited by lack of specificity and potential toxicity [20,21,42].

These limitations have prompted the search for alternative modulators, including natural compounds such as curcumin, resveratrol, and piperine, which may act on multiple targets with potentially improved safety profiles [6,22,42].

An additional layer of epigenetic regulation involves redox signaling. Reactive oxygen species (ROS) can influence the activity of epigenetic enzymes such as DNMTs, TETs, and HDACs, alter the availability of key metabolic cofactors including S-adenosylmethionine (SAM) and α-ketoglutarate, and induce oxidative DNA damage (e.g., 8-OHdG), thereby compromising methylation fidelity [4,5]. Conversely, epigenetic mechanisms regulate antioxidant pathways, including NRF2, SOD1, and GPx, establishing a bidirectional feedback loop between oxidative stress and gene regulation [43,44]. These interconnected regulatory processes converge in the control of cellular plasticity. Epigenetic reprogramming enables cells to transition between differentiated and stem-like states, contributing to tumor progression, metastasis, and therapeutic resistance. In chronic inflammatory diseases, similar mechanisms drive persistent pathological activation of immune and stromal cells [45,46].

While this plasticity underlies disease progression, it also represents a potential therapeutic vulnerability, as epigenetic states can be reprogrammed through targeted or multi-target interventions [47,48,49] (Table 1).

## 5. Diet, Microbiota, and the Epigenome

Another critical axis in the epigenetic modulation of chronic diseases is diet. Nutrients such as folate, methionine, vitamin B12, and polyphenols directly affect the availability of methyl groups and the activity of epigenetic enzymes. Additionally, the gut microbiota influences the host epigenome through the production of short-chain fatty acids such as butyrate, which inhibit HDACs and regulate the expression of anti-inflammatory genes [3,52,53,54].

This has led to the emergence of the field of nutriepigenetics, which aims to identify dietary patterns capable of modulating epigenetic disease risk from early stages of life, including during fetal development (intrauterine epigenetic programming) [55,56].

In cancer and multiple chronic conditions, epigenetic alterations are key drivers of gene program deregulation, loss of cellular identity, evasion of apoptosis, and acquisition of aggressive or therapy-resistant phenotypes. This understanding has elevated epigenetics to a high-value therapeutic target, not only for pharmacological approaches but also for strategies involving natural compounds and lifestyle interventions [57,58].

The reversibility of epigenetic marks makes epigenetics a powerful tool for modulating disease biology, opening avenues for the development of personalized, less toxic, and potentially preventive therapies—particularly in populations with high genetic or environmental risk [59,60]. Thus, integrating epigenetic knowledge into the design of therapeutic and preventive interventions represents one of the greatest challenges and promises of 21st-century medicine.

Given the central role of epigenetic dysregulation in cancer and chronic diseases, considerable efforts have been directed toward identifying compounds capable of modulating these processes in a reversible and context-dependent manner [1,3]. While synthetic epigenetic drugs have demonstrated clinical efficacy, particularly DNA methyltransferase and histone deacetylase inhibitors, their limitations—including lack of specificity and associated toxicities—have prompted increasing interest in naturally derived bioactive molecules [20,21]. Among these, dietary alkaloids have emerged as potential modulators of epigenetic and transcriptional networks, with reported effects on DNA methylation, histone modification, and non-coding RNA regulation [6,22].

However, it is important to note that not all bioactive compounds exert direct or specific epigenetic effects. In many cases, their activity reflects indirect modulation of interconnected pathways, including redox balance, signaling cascades, and metabolic regulation. This distinction is critical when interpreting the biological relevance of these compounds and helps to avoid overestimating their mechanistic specificity.

Within this context, piperine—the principal alkaloid of *Piper nigrum*—has attracted growing attention due to its reported ability to influence multiple regulatory layers, including DNA methylation, histone modification, microRNA expression, and oxidative stress [61]. Nevertheless, the available evidence remains heterogeneous and, in some cases, limited to specific experimental models. Therefore, a critical and integrative analysis of piperine’s mechanisms of action is required to clarify its role as a potential epigenetic modulator.

## 6. The Importance of Dietary Alkaloids as Epigenetic Modulators

Over recent decades, the understanding of how diet influences health has evolved from a purely nutritional framework to a molecular and epigenetic perspective. In this context, dietary alkaloids, nitrogen-containing plant-derived compounds traditionally associated with pharmacological properties—have gained increasing attention as natural epigenetic modulators [62,63]. These compounds, present in spices, infusions, functional foods, and nutraceuticals, possess the ability to reversibly alter gene expression through epigenetic mechanisms without modifying the DNA sequence [64,65]. This positions them as key agents in the prevention, treatment, and potential reversal of non-communicable chronic diseases, including cancer, neurodegenerative, cardiovascular, and metabolic disorders [66,67,68,69].

The main epigenetic mechanisms regulated by dietary alkaloids include DNA methylation, post-translational histone modifications, microRNA expression, and chromatin remodeling [3,25,70]. These pathways are essential for maintaining cellular identity and function, and their dysregulation is implicated in the progression of numerous diseases. Unlike synthetic epigenetic agents, natural compounds such as alkaloids offer a less toxic alternative with multi-target actions, making them promising candidates for both therapeutic and preventive interventions [3,18,70,71,72].

One of the most extensively studied examples is piperine, the principal alkaloid of Piper nigrum (black pepper), which has demonstrated regulatory effects on DNA methylation and the expression of microRNAs associated with cell proliferation, apoptosis, and migration [61,73]. In cancer models, piperine has been shown to reduce the expression of DNA methyltransferases such as DNMT3B and to increase the expression of tumor-suppressor microRNAs such as miR-29c [13]. This dual effect suggests a coordinated epigenetic mechanism contributing to oncogene suppression and reactivation of silenced genes. Additionally, piperine can stabilize G-quadruplex structures in the promoters of oncogenes such as c-MYC, interfering with the transcription of key genes involved in tumor proliferation [14].

Other relevant alkaloids include berberine, present in species of the genus Berberis, which has demonstrated effects on hypermethylation of tumor suppressor gene promoters [74]; capsaicin, the spicy component of chili peppers, which can alter histone acetylation profiles and the expression of apoptotic genes [75]; and nicotine, which, despite its known adverse effects, also modulates epigenetic pathways related to neuroplasticity and addiction [76].

A particularly attractive feature of alkaloids is their capacity to act as dual modulators, exerting opposing effects depending on dose and physiological context [77]. For example, some alkaloids function as antioxidants and regulators of gene expression [78] while others can induce oxidative stress by increasing ROS and activate apoptotic pathways, which is desirable in the context of tumors [79,80]. This versatility should be carefully considered when designing therapeutic strategies or long-term supplementation.

From an epigenetic standpoint, the action of these compounds can be understood as part of a functional reprogramming process, wherein cells are induced to modify their identity and behavior without requiring genetic alterations. In this sense, dietary alkaloids enable intervention in complex processes such as chronic inflammation, insulin resistance, tumor angiogenesis [81], or neurodegeneration through coordinated regulation of multiple gene targets [82]. Moreover, as part of the diet, these compounds have strong potential for inclusion in preventive medicine strategies, particularly in at-risk populations.

In terms of public health and the development of accessible therapies, the study of alkaloids as epigenetic modulators represents a unique opportunity. Their natural availability, relatively low cost, and the possibility of formulation into functional foods, capsules, or standardized extracts make them viable tools in both conventional healthcare systems and traditional or complementary medicine [83].

Nonetheless, several key challenges remain: most available studies are preclinical, and their effects must be validated in robust clinical models. Additionally, effective and safe long-term doses must be established, and epigenetic biomarkers are needed to monitor the efficacy of interventions. The integration of techniques such as global DNA methylation profiling, microRNA expression analysis, and transcriptomics in response to alkaloid treatments will be essential for advancing toward personalized and epigenetically informed medicine [84].

Dietary alkaloids represent a promising class of bioactive compounds with considerable potential as natural modulators of the epigenome [85]. Current research in this area deepens our understanding of the link between nutritional intake and gene regulation, while also revealing new therapeutic strategies to address complex diseases. This line of research supports a more holistic approach to health, connecting dietary patterns, epigenetic mechanisms, and molecular medicine within a unified framework.

## 7. Chemical Nature, Pharmacokinetics, and Bioavailability of Piperine

**Figure 1:**
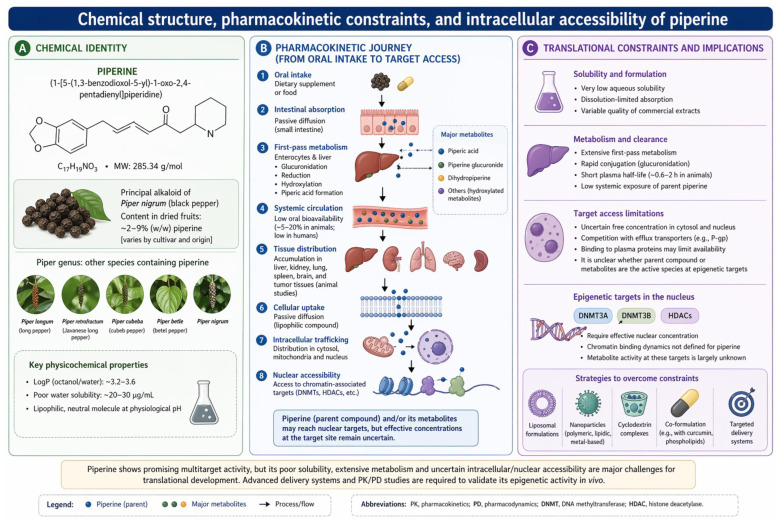
Chemical structure, pharmacokinetic profile, and translational constraints of piperine. The figure illustrates piperine’s chemical identity (**A**), tracing its pharmacokinetic journey from oral intake to potential nuclear accessibility (**B**), and highlighting the key translational challenges that limit its clinical application, including poor aqueous solubility, rapid first-pass metabolism, and uncertain intracellular concentrations at epigenetic targets. Panel (**C**) presents formulation-based strategies currently explored to address these barriers. DNMT, DNA methyltransferase; HDAC, histone deacetylase; PK, pharmacokinetics; PD, pharmacodynamics.

Piperine, the principal pungent alkaloid of *Piper nigrum* and *Piper longum*, is a hydrophobic amide compound with the molecular formula C_17_H_19_NO_3_ and a molecular weight of 285.34 g/mol. Structurally, piperine consists of a piperidine ring linked to a methylenedioxyphenyl moiety through a conjugated aliphatic chain, a configuration that contributes to its lipophilicity and potential interaction with biological membranes and intracellular targets [9,10]. These physicochemical properties have been associated with the broad biological activity of piperine, including its capacity to influence inflammatory, oxidative, metabolic, and transcription-associated pathways (Figure 1).

Despite its promising biological effects, piperine exhibits important pharmacokinetic limitations that may influence its therapeutic applicability. Piperine has low aqueous solubility and undergoes extensive metabolic transformation, factors that can restrict systemic bioavailability and tissue distribution. Nevertheless, experimental studies have demonstrated gastrointestinal absorption, plasma detection, and tissue distribution following oral and intravenous administration [12]. In addition, piperine has been reported to modulate intestinal permeability and drug-metabolizing enzymes, thereby increasing the bioavailability of several compounds, including curcumin and carbamazepine [11,86,87]. These effects have been attributed, at least in part, to interactions with cytochrome P450 enzymes and efflux transporters involved in xenobiotic metabolism.

Importantly, although piperine has demonstrated biological activity in multiple experimental models, its intracellular pharmacodynamics remain incompletely understood. In particular, whether piperine or its metabolites reach nuclear compartments at concentrations sufficient to directly modulate epigenetic enzymes, chromatin-associated complexes, or transcriptional regulators remains uncertain. Therefore, the interpretation of piperine as a direct epigenetic modulator should be approached cautiously, especially considering the heterogeneity of experimental systems, concentrations, formulations, and exposure conditions reported across studies.

## 8. Piperine as an Integrated Modulator of MicroRNA Expression and DNA Methylation

MicroRNAs (miRNAs) have emerged as critical post-transcriptional regulators of gene expression, participating in a broad range of physiological and pathological processes, including cancer, fibrosis, cardiovascular disorders, and chronic pain. These small non-coding RNAs (~22 nucleotides) act by inhibiting translation or promoting degradation of target mRNAs, thereby fine-tuning protein synthesis [88,89,90]. Dysregulation of miRNAs has been implicated in disease pathogenesis, prompting significant interest in natural compounds capable of modulating miRNA networks. Among these, piperine—a dietary alkaloid from Piper nigrum—has shown notable bioactive and epigenetic properties [73]. One of the best-characterized examples is miR-29c, a tumor-suppressive miRNA known to inhibit DNMT3A and DNMT3B, key DNA methyltransferases involved in epigenetic silencing of tumor suppressor genes. In triple-negative breast cancer and hepatocellular carcinoma cell lines, piperine has been shown to increase miR-29c expression while simultaneously downregulating DNMT3B, leading to promoter demethylation and reactivation of silenced genes [13]. This dual action highlights piperine’s capacity to intervene at both post-transcriptional and transcriptional levels, offering coordinated control of epigenetic reprogramming.

Beyond carcinogenesis, piperine’s modulation of miRNA expression extends to other disease contexts. In hepatic fibrosis, piperine reduced miR-17-5p—an oncomiR associated with TGF-β/Smad pathway activation, restoring Smad7 levels and inhibiting collagen deposition [91]. In cancer-related pain models, piperine increased miR-150-5p expression, suppressing microglial activation and proinflammatory mediators such as CXCL2 and Iba1 [92]. In the cardiovascular setting, piperine decreased miR-383 in myocardial ischemia-reperfusion injury models, leading to reduced pyroptosis and restoration of PI3K/AKT signaling [93]. Parallel to these miRNA-mediated effects, piperine exerts direct epigenetic influence through modulation of DNA methylation. DNA methylation, catalyzed by DNMT1, DNMT3A, and DNMT3B, is one of the most widely studied epigenetic marks, frequently dysregulated in cancer. Overexpression of DNMT3B contributes to silencing of tumor suppressor genes and acquisition of malignant phenotypes. Piperine’s inhibition of DNMT3B, demonstrated at both mRNA and functional levels—translates into demethylation of CpG islands in promoter regions and restoration of key cellular functions such as apoptosis, cell cycle regulation, and DNA repair [13]. Importantly, piperine appears to selectively target tumor cells without altering methylation patterns in non-transformed tissues [94], a property that enhances its therapeutic potential while minimizing off-target effects. Its daily dietary intake further raises the possibility of long-term chemopreventive effects through cumulative epigenetic modulation, positioning piperine as a promising agent in the emerging field of nutriepigenetics. Taken together, current evidence suggests that piperine functions as an integrated epigenetic modulator capable of reprogramming gene expression through both miRNA regulation and direct interference with the DNA methylation machinery. This multifocal activity underscores its therapeutic versatility, supporting its potential inclusion in targeted epigenetic therapies and nutraceutical interventions for cancer and other complex diseases.

## 9. Piperine and G-Quadruplex Stabilization

In the complex architecture of the human genome, the structure of DNA extends beyond the classic double helix proposed by Watson and Crick. Alternative DNA conformations, known as non-canonical secondary structures, play essential regulatory roles. Prominent among these are G-quadruplexes (G4s), four-stranded structures stabilized by stacked guanine tetrads held together by Hoogsteen hydrogen bonds. These tetrads form planar, stable structures by coordinating with monovalent cations such as potassium (K^+^). G4s can form in both DNA and RNA, especially in promoter regions, telomeric sequences, and non-coding regulatory elements [95].

G-quadruplexes are not merely structural curiosities but functional elements involved in the regulation of transcription, replication, genome stability, and translation. Their presence in the promoters of oncogenes such as c-MYC, c-KIT, BCL2, and KRAS has attracted considerable scientific interest, as G4 stabilization impedes transcriptional machinery progression, thereby reducing gene expression. Consequently, G4s have emerged as unconventional therapeutic targets that can be modulated by small molecules capable of inducing or stabilizing their formation [96,97,98].

Preliminary evidence suggests that piperine may interact with these structures. In particular, a single biophysical study by Tawani and colleagues demonstrated that piperine stabilizes the G4 motif within the c-MYC promoter, resulting in reduced transcriptional activity and induction of apoptosis in cancer cells [14]. While these findings open an intriguing perspective on piperine’s structural interactions with the genome, this mechanism remains largely unexplored and requires independent validation in additional models and genomic contexts. Thus, the ability of piperine to modulate gene expression through G-quadruplex stabilization should currently be considered as an emerging hypothesis rather than a consolidated mechanism, underscoring the need for further in vitro, in vivo, and genome-wide studies [14].

This structural interaction has significant functional implications. Stabilization of the G-quadruplex within the c-MYC promoter hinders the binding of essential transcription factors, leading to reduced transcription and protein expression. c-MYC encodes a master transcription factor involved in the regulation of the cell cycle, proliferation, biosynthesis, and cellular metabolism [99]. Its overexpression is associated with unchecked growth, resistance to apoptosis, and malignant transformation. Therefore, its silencing via non-genetic mechanisms represents an attractive strategy for antitumor therapy development.

Piperine’s effects on the c-MYC pathway are not limited to transcriptional inhibition. In the same study, researchers also observed decreased cell viability, DNA fragmentation, and apoptosis induction in both cancer cell lines and experimental models. These outcomes were validated using TUNEL assays, gel electrophoresis, and RT-PCR gene expression analyses. Collectively, the findings suggest that piperine’s action on G-quadruplex structures is not purely structural but translates into direct biological consequences that drive programmed cell death in cancer cells, positioning it as a molecule with multiple and convergent anticancer properties [14].

What is particularly notable about this mechanism is that it differs from the classical epigenetic effects of piperine on DNMTs or miRNAs. Here, the action is not on enzyme expression or post-transcriptional processing but rather on the three-dimensional architecture of regulatory DNA itself, altering its accessibility to transcriptional activation. This layer of regulation highlights the functional versatility of piperine and its potential to impact multiple levels of gene control.

Moreover, the implications of G-quadruplex stabilization extend beyond c-MYC. Numerous telomeric regions, pluripotency-associated genes, repetitive elements, and transposable elements contain sequences capable of forming G4s. Although the effect of piperine on all such sites has not yet been fully characterized, its affinity for G4 structures suggests that it could modulate entire gene networks, should similar interactions be confirmed across other genomic contexts. This opens a new avenue of research on the use of piperine as a structural modulator of the functional genome, with potential applications in oncology, aging, and genetic diseases [100].

In summary, piperine’s ability to stabilize G-quadruplexes represents an emerging and innovative mechanism of epigenetic action. Through its interaction with secondary structures in promoter regions such as that of the c-MYC oncogenepiperine can induce negative regulation of key genes involved in tumor proliferation, promote apoptosis, and reduce cancer cell viability. This pharmacologically underexplored pathway represents a valuable opportunity for developing natural agents capable of directly reshaping the regulatory architecture of the genome and, in doing so, reprogramming the fate of cancer cells.

## 10. Piperine and Transcriptional Pathways

In addition to its epigenetic effects on DNA methylation and microRNA regulation, piperine has shown remarkable capacity to modulate transcriptional signaling pathways that govern fundamental processes such as cell proliferation, migration, tumor invasion, and differentiation. These pathways act as functional hubs that integrate microenvironmental signals and determine cell fate; thus, their regulation represents a high-impact therapeutic strategy in diseases like cancer [101,102,103]. Among the best-characterized signaling cascades modulated by piperine are the Wnt/β-catenin pathway, the PI3K/AKT/mTOR axis, and the STAT3/Snail-mediated epithelial–mesenchymal transition (EMT) signaling.

The Wnt/β-catenin pathway is essential for tissue homeostasis, stem cell renewal, and embryogenesis. However, its aberrant activation is associated with the initiation and progression of various cancers. In the absence of Wnt signaling, β-catenin is phosphorylated and targeted for degradation. When the pathway is activated, β-catenin becomes stabilized, translocates into the nucleus, and activates genes such as c-MYC, cyclin D1, and survivin—all promoters of proliferation and cell survival. A pioneering study demonstrated that piperine inhibits Wnt signaling in normal mammary stem cells and mammospheres treated with piperine alone or in combination with curcumin [16]. Using the TopFlash reporter system, which employs GFP under the control of the TCF/LEF promoter, the authors observed a significant reduction in β-catenin–mediated transcriptional activity. This was accompanied by a decrease in mammosphere size, suggesting a loss of self-renewal potential [16]. Subsequently, other study confirmed these findings in colorectal cancer cell lines, where piperine blocked β-catenin nuclear translocation, reduced c-MYC expression, and inhibited proliferation without affecting non-tumor cells reinforcing its selective action [104].

Another key pathway modulated by piperine is the PI3K/AKT/mTOR axis, a central regulator of cell survival, metabolism, angiogenesis, and metastasis. PI3K activation leads to phosphorylation of AKT, which in turn activates mTOR, thereby promoting cell growth and protein synthesis. In pathological conditions such as prostate cancer, this pathway is often hyperactivated, contributing to tumor invasiveness [105]. A study showed that piperine treatment reduced levels of AKT and mTOR, as well as the expression of MMP-9 an essential metalloproteinase for extracellular matrix degradation and cell migration [17]. These findings suggest that piperine disrupts pro-metastatic signaling, thereby limiting the invasive potential of tumor cells. Furthermore, the coordinated inhibition of these components implies that piperine may act upstream in the PI3K pathway or directly on AKT, although this warrants further molecular characterization [17].

Finally, piperine also influences the regulation of epithelial–mesenchymal transition (EMT), a process through which epithelial cells lose polarity and adhesion properties while acquiring mesenchymal traits associated with migration, invasion, stemness, and apoptosis resistance. EMT is considered a critical event in tumor progression and metastatic dissemination. Experimental studies have demonstrated that piperine suppresses EMT-associated phenotypes through modulation of interconnected transcriptional pathways, including STAT3/Snail, Wnt/β-catenin, and PI3K/AKT signaling [15,17]. In colorectal cancer models, piperine reduced expression of the transcription factor Snail, restored E-cadherin expression, and decreased levels of the mesenchymal marker vimentin, indicating partial reversal of the mesenchymal phenotype [106]. Additionally, inhibition of STAT3 phosphorylation was associated with reduced migration and invasion capacity, reinforcing the role of piperine as a modulator of EMT-associated transcriptional programs [106]. Given the close relationship between EMT, stemness, and therapeutic resistance, these findings further support the potential relevance of piperine in regulating tumor plasticity and cancer stem cell-associated phenotypes [16].

These findings indicate that piperine does not act through a single molecular target, but rather modulates multiple interconnected transcriptional regulatory nodes converging on key processes such as proliferation, invasion, epithelial–mesenchymal transition (EMT), stemness, and cellular plasticity [15,16,17]. This multitarget activity may enhance its therapeutic relevance, since simultaneous modulation of complementary signaling pathways could reduce compensatory mechanisms frequently associated with therapeutic resistance. Unlike highly selective agents directed against a single protein, piperine appears to influence broader regulatory networks involving oxidative stress responses, oncogenic signaling, and transcription-associated pathways [103,106].

Collectively, current evidence indicates that piperine modulates several aberrant transcriptional pathways in cancer, including Wnt/β-catenin signaling, the PI3K/AKT/mTOR axis, NF-κB-associated inflammatory signaling, and EMT-related pathways mediated through STAT3/Snail regulation [15,17,106]. Its capacity to downregulate oncogenic transcriptional programs, suppress migration- and invasion-associated factors, and partially restore epithelial identity suggests that its biological effects extend beyond isolated epigenetic modulation and may involve broader reprogramming of tumor-associated cellular behavior. These observations position piperine as a pleiotropic bioactive compound with potential relevance for the development of multitarget therapeutic strategies against aggressive and treatment-resistant cancers.

## 11. Piperine-Induced Apoptosis: Epigenetic and Redox-Dependent Mechanisms

**Figure 2:**
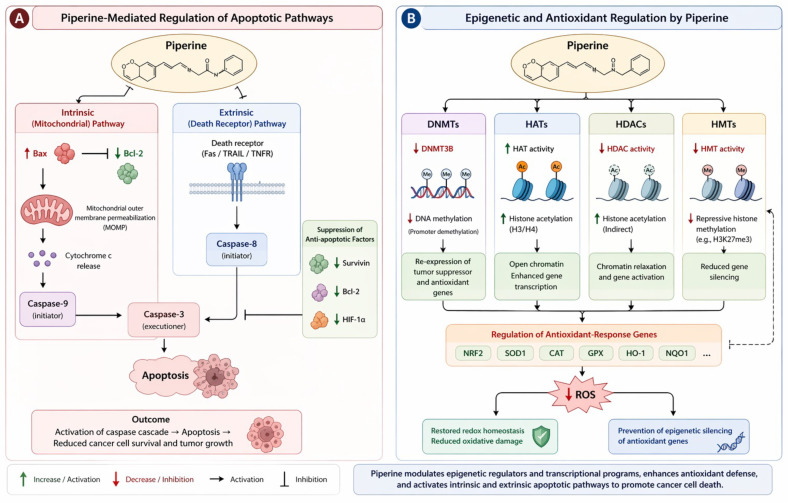
Piperine-mediated regulation of apoptotic pathways and epigenetic control of antioxidant response in cancer cells. Panel (**A**) illustrates how piperine engages both the intrinsic (mitochondrial) and extrinsic (death receptor) apoptotic pathways, driving Caspase-3-dependent cancer cell death through Bax/Bcl-2 imbalance, cytochrome c release, and suppression of key survival factors including Survivin, Bcl-2, and HIF-1α. Panel (**B**) shows how piperine reshapes the epigenetic landscape of cancer cells by reducing DNMT3B activity and repressive histone methylation (H3K27me3) while promoting histone acetylation, effects that collectively restore expression of tumor suppressor and antioxidant genes, enhance NRF2 pathway activity, and lower intracellular ROS levels. DNMT, DNA methyltransferase; HAT, histone acetyltransferase; HDAC, histone deacetylase; HMT, histone methyltransferase; ROS, reactive oxygen species.

Evasion of apoptosis is a hallmark of cancer and represents a major obstacle to effective therapy, particularly in aggressive and treatment-resistant tumors. In this context, increasing evidence suggests that piperine may exert anticancer activity not only through inhibition of proliferative signaling pathways, but also through coordinated modulation of apoptosis-related networks involving mitochondrial dysfunction, oxidative stress, epigenetic remodeling, and transcriptional regulation. Importantly, the pro-apoptotic effects associated with piperine appear to be highly context-dependent and frequently intersect with redox-sensitive and epigenetic mechanisms rather than acting through a single isolated pathway (Figure 2).

One of the most consistently reported effects of piperine in cancer models is the activation of mitochondrial apoptosis. Experimental studies in breast, colorectal, prostate, gastric, and leukemia-related cancer models have shown that piperine promotes mitochondrial membrane depolarization, cytochrome c release, and activation of the intrinsic caspase cascade, including Caspase-9 and Caspase-3, together with increased expression of the pro-apoptotic protein Bax and suppression of anti-apoptotic mediators such as Bcl-2 and surviving [103,106,107].

These molecular events ultimately contribute to DNA fragmentation, loss of cell viability, and apoptotic cell death. In addition, recent evidence in acute leukemia models demonstrated that piperine-induced apoptosis may also involve extracellular vesicle-mediated mechanisms associated with increased cathepsin D (CTSD), Bax, and Caspase-3 expression, suggesting that piperine can modulate intercellular signaling pathways linked to apoptosis propagation [108].

Accumulating evidence indicates that oxidative stress plays a central role in these apoptotic responses. Depending on concentration and cellular context, piperine may function either as an antioxidant or as a pro-oxidant compound capable of inducing reactive oxygen species (ROS) accumulation and mitochondrial dysfunction [103,109]. Additional mechanistic evidence suggests that piperine-induced apoptosis may involve modulation of mitochondrial-associated apoptotic regulators, including PARP-1 cleavage, XIAP suppression, and stress-responsive JNK/p38 MAPK signaling [103,110]. In HER2-positive breast cancer and melanoma models, piperine has been associated with suppression of anti-apoptotic proteins and facilitation of caspase-dependent apoptotic signaling, supporting the predominance of intrinsic mitochondrial apoptosis pathways. Furthermore, ROS-dependent mitochondrial depolarization and DNA fragmentation have been consistently observed across multiple cancer models, reinforcing the role of oxidative stress as a central mediator of piperine-associated cytotoxicity [103,107]. In tumor cells, elevated ROS generation following piperine exposure has been associated with mitochondrial permeability transition, ATP depletion, oxidative DNA damage, and activation of stress-responsive apoptotic signaling pathways [107]. Importantly, oxidative stress may also influence epigenetic machinery by altering the activity of DNMTs, HDACs, and redox-sensitive transcription factors, thereby creating a mechanistic bridge between ROS accumulation and epigenetic reprogramming. Importantly, piperine exhibits a context-dependent redox duality, functioning as an antioxidant at lower concentrations while promoting pro-oxidant cytotoxicity at higher doses, particularly in tumor-associated experimental systems [103,109]. At low concentrations, piperine acts as a potent ROS scavenger, mitigating oxidative damage; however, at higher concentrations, it shifts toward a pro-oxidant profile, increasing free radical generation and triggering oxidative stress-mediated apoptosis in cancer cells [103]. This pro-oxidant activity has been mechanistically substantiated in experimental models: in a diethylnitrosamine-induced hepatocellular carcinoma rat model, the antitumor efficacy of piperine was significantly attenuated by antioxidant co-treatment, providing in vivo evidence that its anticancer action is mediated, at least in part, through oxidative mechanisms [111]. This shift appears especially relevant in cancer cells, which frequently exhibit elevated basal ROS levels due to increased metabolic activity, mitochondrial dysfunction, and oncogene activation [112,113]. The resulting state of chronic oxidative stress, while initially advantageous for tumor progression, paradoxically renders cancer cells highly susceptible to further ROS-inducing insults, as any additional pro-oxidant burden may rapidly exceed the cytotoxic threshold [103,114].

Emerging evidence further suggests that piperine-associated apoptosis may be linked to epigenetic and transcriptional regulation. In cancer models, piperine has been reported to increase expression of the tumor suppressor miR-29c while simultaneously decreasing DNMT3B levels, a mechanism associated with altered transcriptional regulation and potential reactivation of tumor suppressor pathways [13]. Since aberrant DNMT3B expression contributes to apoptosis resistance and epigenetic silencing of pro-apoptotic genes, these findings raise the possibility that piperine-induced apoptosis may occur, at least partially, through epigenetic derepression mechanisms. Likewise, suppression of oncogenic signaling pathways such as PI3K/AKT/mTOR, Wnt/β-catenin, and STAT3/Snail by piperine may further sensitize tumor cells to apoptosis by reducing survival signaling, EMT-associated plasticity, and resistance-related transcriptional programs [15,16,17,106]. Additional evidence indicates that piperine may influence hypoxia- and stress-associated transcriptional regulators involved in apoptosis resistance. Experimental studies have reported reduced HIF-1α expression following piperine treatment, together with suppression of downstream survival and angiogenesis-associated pathways [103].

Moreover, piperine-mediated inhibition of NF-κB signaling has been associated with decreased inflammatory and anti-apoptotic transcriptional activity, including reduced expression of Bcl-2-associated survival programs. Specifically, piperine has been shown to block NF-κB nuclear localization and suppress phosphorylated STAT-3, leading to downregulation of Bcl-2 and a consequent increase in the Bax:Bcl-2 ratio that favors caspase-9/3-dependent apoptotic execution [46,103,115]. Collectively, these observations support the concept that piperine-associated apoptosis is not exclusively mediated by direct mitochondrial injury, but rather emerges from integrated interactions between oxidative stress, epigenetic remodeling, and transcriptional regulation—a multi-targeted mechanistic profile consistent with its broad anticancer activity across diverse tumor models [13,103,116].

Taken together, current evidence suggests that piperine may promote apoptosis through a multifactorial and interconnected regulatory network involving ROS accumulation, mitochondrial dysfunction, microRNA modulation, DNA methylation-associated pathways, and suppression of oncogenic transcriptional signaling. However, despite these promising findings, the mechanistic evidence remains heterogeneous and largely restricted to preclinical models. Therefore, additional studies are required to clarify the direct molecular targets of piperine, determine the relative contribution of epigenetic versus redox-dependent mechanisms, and establish whether these effects can be translated into clinically relevant therapeutic strategies.

## 12. Discussion

The accumulated evidence on piperine reveals that this alkaloid does not act through a single mechanism, but rather exerts coordinated effects across multiple levels of cellular regulation, including epigenetic modifications, transcriptional signaling pathways, and intracellular redox status. Its ability to modulate different functional nodes raises fundamental questions about the molecular specificity of piperine, its actual therapeutic value, and how it compares to other bioactive dietary compounds (Table 2).

One of the most intriguing characteristics of piperine is its capacity to generate functional crosstalk between cellular redox balance, epigenetic modifiers, and signaling pathways. For instance, in gastric cancer models, piperine has been shown to induce oxidative stress by increasing the production of reactive oxygen species (ROS), leading to mitochondrial apoptosis. However, this ROS increase may also indirectly affect the activity of epigenetic enzymes such as DNMTs, TETs, and HDACs, which are highly dependent on redox balance and redox-sensitive cofactors. In this way, piperine acts not only as a direct prooxidant in tumor cells but also as an inducer of secondary epigenetic reconfiguration, favoring the re-expression of pro-apoptotic or tumor suppressor genes that were previously silenced.

This molecular dialog extends to the regulation of microRNAs as well. Piperine has been shown to induce or repress the expression of several functional miRNAs (e.g., miR-29c, miR-383, miR-150-5p), which in turn regulate both epigenetic enzymes (e.g., DNMT3B) and components of signaling pathways (e.g., PI3K, Smad7). This phenomenon suggests that piperine integrates multiple layers of cellular regulation, acting as a "bioactive hub" capable of reshaping cell behavior from its epigenetic architecture to its transcriptional and biochemical responses.

This multi-activity inevitably raises the question of specificity versus pleiotropy. In classical pharmacology, molecular specificity is often regarded as a desirable trait. However, in the context of natural compounds with multi-target therapeutic applications such as piperine, pleiotropy may represent an advantage, particularly in complex diseases like cancer, where resistance mechanisms often rely on molecular redundancy. Piperine exhibits moderate affinity for structural targets (e.g., G-quadruplexes), modulates miRNA expression in a context-dependent manner, and regulates key transcriptional pathways such as Wnt/β-catenin and PI3K/AKT/mTOR. This profile suggests that its activity is not driven by high-affinity binding to a single target, but rather by a synergistic influence over cellular networks, potentially resulting in functional re-equilibration in diseased tissues.

Compared to other dietary epigenetic modulators such as curcumin, resveratrol, or epigallocatechin gallate (EGCG) piperine exhibits both similarities and unique features. Like these compounds, it has limited bioavailability, which has prompted investigations into liposomal formulations, nanoparticles, and synergistic combinations (e.g., with curcumin). It also shares the ability to modulate DNMTs, HDACs, miRNAs, and signaling pathways. Evidence from a single study suggests that piperine could stabilize the c-MYC promoter G-quadruplex; however, this mechanism remains preliminary and requires further validation in diverse models. Clarifying its role will be essential to determine whether G-quadruplex stabilization represents a consistent or context-dependent facet of piperine’s activity. Despite promising preclinical findings, the translational applicability of piperine remains limited by poor aqueous solubility, low bioavailability, rapid metabolism, and the scarcity of clinical evidence.

Another distinctive feature is its context-dependent activity, which may confer a degree of therapeutic selectivity. For instance, the downregulation of DNMT3B and induction of miR-29c are observed in tumor cell lines, while no deleterious effects have been reported in non-transformed cells. This suggests that its effects may be conditioned by the cellular microenvironment, the basal expression of its targets, or the pre-existing epigenetic landscape—an especially desirable property for agents aimed at chemoprevention.

From a clinical perspective, piperine’s multilayered effects make it a candidate for use both as a chemosensitizing agent—enhancing the efficacy of conventional drugs—and as part of combinatorial nutraceutical regimens targeting complementary pathways. Given its relative safety, its inclusion in preventive strategies, particularly in at-risk populations or individuals with predisposing mutations, constitutes a research avenue of high priority.

Recent studies on piperine-derived compounds suggest that structural optimization may enhance the ability of this chemical scaffold to modulate transcriptional programs involved in cancer progression. For example, the piperine derivative HJ-4 suppressed colorectal tumor growth and angiogenesis while activating p53-dependent apoptosis and inhibiting Wnt/β-catenin and E2F transcriptional activity [117]. Similarly, HJJ_3_5 inhibited colorectal cancer migration and invasion through SNAI1-mediated EMT [118]. Although these findings cannot be directly extrapolated to native piperine, they support the relevance of the piperine scaffold as a platform for designing more potent modulators of oncogenic transcriptional networks.

In summary, piperine emerges as a broad-spectrum epigenetic and transcriptional modulator whose effects appear to depend not only on interactions with DNA-binding proteins or structural motifs but also on redox balance and cellular context. While its pleiotropic nature may represent an advantage in addressing complex diseases such as cancer, the current body of evidence remains incomplete and largely preclinical. Critical questions regarding dose–response relationships, long-term safety, bioavailability, and functional effects in advanced models—including cancer stem cells and patient-derived systems—still need to be addressed. When compared to other dietary alkaloids or polyphenols, piperine displays distinctive features; however, only through rigorous in vivo validation and translational studies will its true therapeutic potential as a functional reprogrammer of diseased cells be established.

## 13. Limitations and Future Perspectives

Despite the growing body of evidence supporting the epigenetic and therapeutic potential of piperine, several methodological and experimental limitations persist, hindering its clinical translation and complicating the standardization of its biological effects. These challenges represent both obstacles and opportunities to deepen our understanding of this bioactive compound and to advance its validation as an epigenetic modulator in complex diseases such as cancer.

One of the main limitations identified in the literature is the lack of long-term in vivo studies with standardized dosing. Most preclinical assays have been conducted in short-term cellular or animal models using piperine formulations that vary widely in concentration, route of administration, and exposure time. This methodological heterogeneity prevents the clear establishment of a dose–response curve, which is essential to determine the threshold between its antioxidant, epigenetic, and pro-oxidant effects. Given that piperine exhibits biphasic activity depending on the dose, it is a priority to define a safe and effective therapeutic range that maximizes benefits without inducing adverse effects related to oxidative stress or mitochondrial toxicity.

In this context, there is an urgent need for longitudinal in vivo studies in relevant models that not only assess the biological efficacy of piperine but also evaluate its bioavailability, hepatic metabolism, tissue accumulation, and long-term safety profile. Moreover, pharmaceutical formulation should be optimized to overcome its poor solubility and rapid systemic clearance, through strategies such as lipid nanoparticles, co-formulations with curcumin, or cyclodextrin-based delivery systems. These advances would allow for a more accurate evaluation of the duration and stability of piperine’s epigenetic effects in target tissues.

Another significant gap in current knowledge is the limited functional evidence in cancer stem cell (CSC) models. Although promising effects of piperine have been reported in conventional tumor cell lines and animal models, its specific impact on CSC populations characterized by chemotherapy resistance, self-renewal capacity, and their role in tumor recurrence remains poorly understood. These cells are considered critical therapeutic targets, especially in gastric, colorectal, and breast cancers, were CSCs drive progression and metastasis.

Preliminary studies suggest that piperine may modulate pluripotency-related genes (e.g., OCT4, SOX2, CD44) and pathways involved in self-renewal (e.g., Wnt/β-catenin and PI3K/AKT), but functional validation in advanced models such as spheroids, organoids, or patient-derived primary cultures is still lacking. The implementation of these models would enable assessment not only of direct cytotoxicity, but also of piperine’s ability to induce differentiation, senescence, or loss of stemness markers—offering stronger evidence for its potential in targeted therapies.

Finally, one of the most promising but underexplored avenues is the application of high-throughput omics technologies, such as epigenome and transcriptome sequencing. Most current studies on piperine focus on a limited number of genes or pathways, constraining our understanding of its global cellular impact. However, methods like RRBS (Reduced Representation Bisulfite Sequencing) or WGBS (Whole Genome Bisulfite Sequencing) could map genome-wide DNA methylation changes following piperine exposure, revealing silenced or reactivated genes at the epigenetic level. Similarly, RNA-seq could uncover complete transcriptomic signatures, identifying gene expression networks affected, alterations in cellular identity, and modulation of non-coding regulators such as lncRNAs or miRNAs.

Developing these integrated approaches would enable the construction of an epigenomic-functional profile of piperine’s action, facilitating its comparison with other dietary modulators and strengthening its application in personalized medicine. Additionally, bioinformatics and network analysis could reveal novel molecular targets and help establish biomarkers of therapeutic response or prognosis in patients treated with piperine-containing formulations. Emerging evidence additionally suggests that piperine may influence alternative cell death programs, including autophagy- and ferroptosis-associated pathways, particularly through ROS-dependent mechanisms and PI3K/AKT/mTOR modulation. However, these observations remain mechanistically underdeveloped and require further validation in the context of epigenetic regulation.

In summary, although piperine has shown promise as an epigenetic and transcriptional modulator, its path to clinical application depends on overcoming key limitations: standardizing dosage and exposure duration in in vivo models, functionally validating its effects in cancer stem cell contexts, and implementing omics-based strategies to characterize its global cellular impact. Only with these tools will it be possible to clearly define its scope of action, key molecular targets, and true therapeutic value in complex pathologies such as cancer. Overall, the 2025–2026 literature does not substantially alter the core mechanistic framework discussed in this review; however, it contributes several important emerging concepts, including extracellular vesicle-mediated apoptotic signaling, the relevance of piperine-derived compounds as modulators of transcriptional networks, and the growing integration of redox-sensitive mechanisms with epigenetic and transcription-associated regulation.

## Figures and Tables

**Table 1:** Epigenetic regulators and their functional impact on cancer-related gene expression.

Epigenetic Regulator	Mechanism of Action	Target Genes/Pathways	Functional Outcome in Cancer	References
DNMT1, DNMT3A, DNMT3B	DNA methylation (CpG islands) → transcriptional repression	Tumor suppressor genes (e.g., CDKN2A, MLH1, BRCA1)	Gene silencing, genomic instability, tumor progression	[1,2]
TET enzymes (TET1–3)	DNA demethylation (5 mC → 5 hmC)	Tumor suppressor and differentiation genes	Reactivation of silenced genes; loss linked to cancer progression	[3]
HATs (e.g., p300/CBP)	Histone acetylation → chromatin relaxation	Genes involved in apoptosis, differentiation	Transcriptional activation; tumor suppressor function	[27]
HDACs (Class I–IV)	Histone deacetylation → chromatin condensation	Tumor suppressor genes, cell cycle regulators	Gene repression, proliferation, therapy resistance	[50]
HMTs (e.g., EZH2)	Histone methylation (e.g., H3K27me3) → repression	Tumor suppressor genes	Epigenetic silencing, CSC maintenance	[28]
HDMs (e.g., LSD1, KDMs)	Histone demethylation	Genes regulating proliferation and differentiation	Context-dependent activation/repression	[4]
miRNAs (e.g., miR-29c, miR-21, miR-34a)	Post-transcriptional repression of mRNA	DNMTs, oncogenes, tumor suppressors	Regulation of apoptosis, proliferation, metastasis	[32,51]
lncRNAs (e.g., HOTAIR, MALAT1)	Chromatin remodeling, miRNA sponging	Epigenetic complexes (PRC2), transcriptional regulators	Promotion of metastasis, EMT, therapy resistance	[3]
Chromatin remodeling complexes (e.g., SWI/SNF)	Nucleosome repositioning	Genome-wide transcriptional regulation	Loss leads to altered differentiation and tumorigenesis	[1]
ROS-sensitive epigenetic regulation	Oxidative modification of DNMTs, HDACs, cofactors	NRF2 pathway, antioxidant genes	Epigenetic instability, redox imbalance, apoptosis regulation	[6,43]

**Table 2:** Principal nutriepigenetic compounds and their mechanisms of action.

Compound	Main Epigenetic Targets	Additional Pathways	Reported Advantages	Limitations	Level of Evidence
Piperine (*Piper nigrum*)	↓ DNMT3B, ↓ HDAC activity, ↑ miR-29c, ↓ miR-383, G-quadruplex stabilization (c-MYC, preliminary)	PI3K/AKT, Wnt/β-catenin, STAT3/EMT, ROS-mediated apoptosis	Multi-target, context-dependent selectivity, dual redox profile, preliminary evidence in CSC-like phenotypes	Poor solubility, rapid metabolism, lack of standardized dosing, limited in vivo/CSC validation	Preclinical in vitro and limited animal studies
Curcumin (*Curcuma longa*)	↓ DNMT1, ↓ HDACs, ↑ histone acetylation, modulation of miR-200 and miR-34a	NF-κB, PI3K/AKT, JAK/STAT	Broad anti-inflammatory and epigenetic activity; well studied; synergistic with piperine	Very low bioavailability, unstable at physiological pH, inconsistent clinical results	Extensive preclinical + early clinical
Resveratrol (*Vitis vinifera)*	Modulation of DNMTs and HDACs, ↑ SIRT1 activity, regulation of miR-663	AMPK, PI3K/AKT, MAPK	Strong antioxidant profile, metabolic benefits, multiple disease contexts	Dose-dependent paradoxical effects; poor solubility; variable outcomes in trials	Extensive preclinical + moderate clinical
EGCG (*Camellia sinensis*)	↓ DNMT1/3B, inhibition of HDACs, reactivation of silenced tumor suppressor genes	PI3K/AKT, MAPK, NRF2	Potent DNMT inhibitor, dietary relevance, strong antioxidant	Instability in plasma, low bioavailability, off-target effects at high doses	Extensive preclinical + some clinical

## Data Availability

Not applicable.

## Abbreviations

The following abbreviations are used in this manuscript:

HATs	Histone acetyltransferases
HDACs	Histone deacetylases
CSCs	Cancer Stem Cells
DNMTs	DNA methyltransferases
TET	Ten-eleven translocation methylcytosine dioxygenases
ROS	Reactive oxygen species
